# Supplementary material for: Identification and Characterization of NPR1 and PR1 Homologs in Cymbidium orchids in Response to Multiple Hormones, Salinity and Viral Stresses
Source: Int J Mol Sci. 2020 Mar 13;21(6):1977. doi: 10.3390/ijms21061977 (PMC7139473; doi:10.3390/ijms21061977)
Supplement: Supplementary file 1 [file ijms-21-01977-s001.pdf]

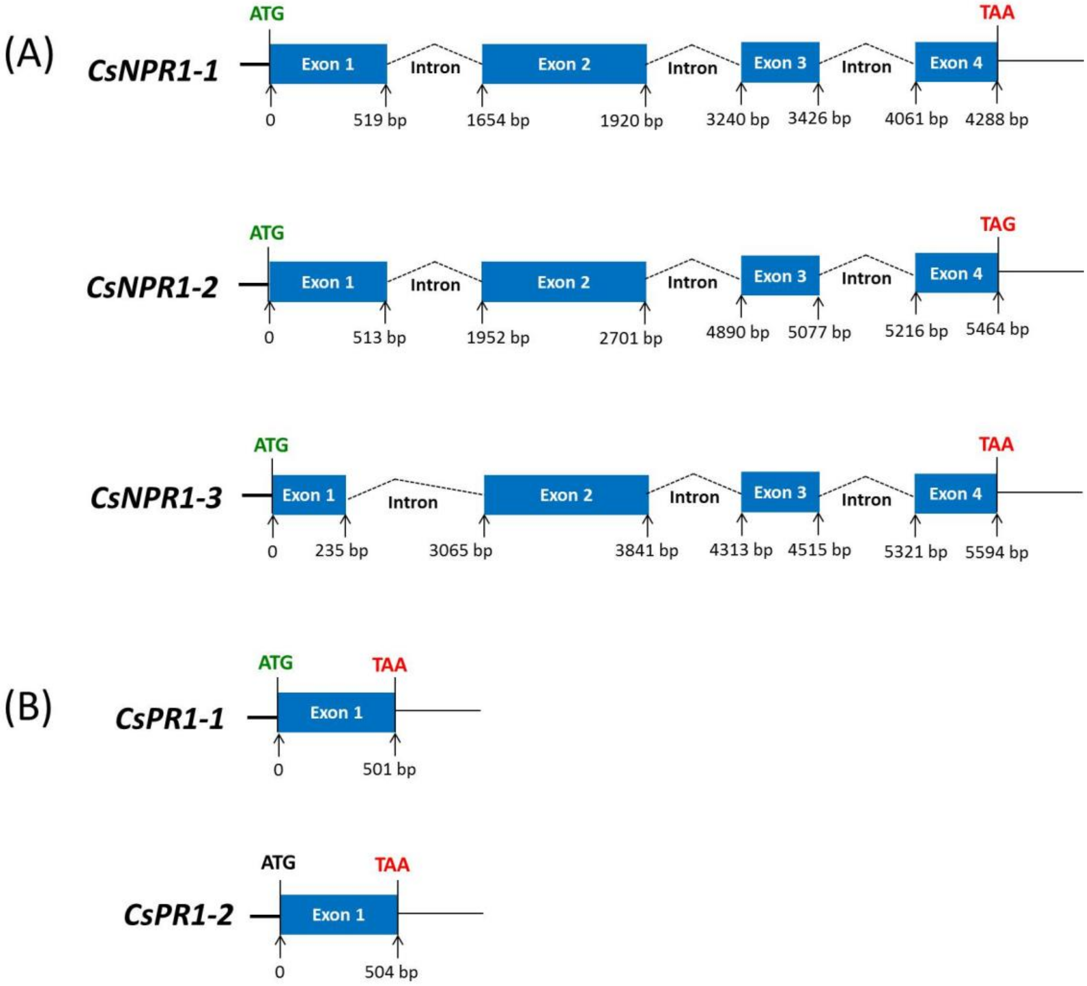

Figure S1: Transcription models of CsNPR1 and CsPR1 genes.

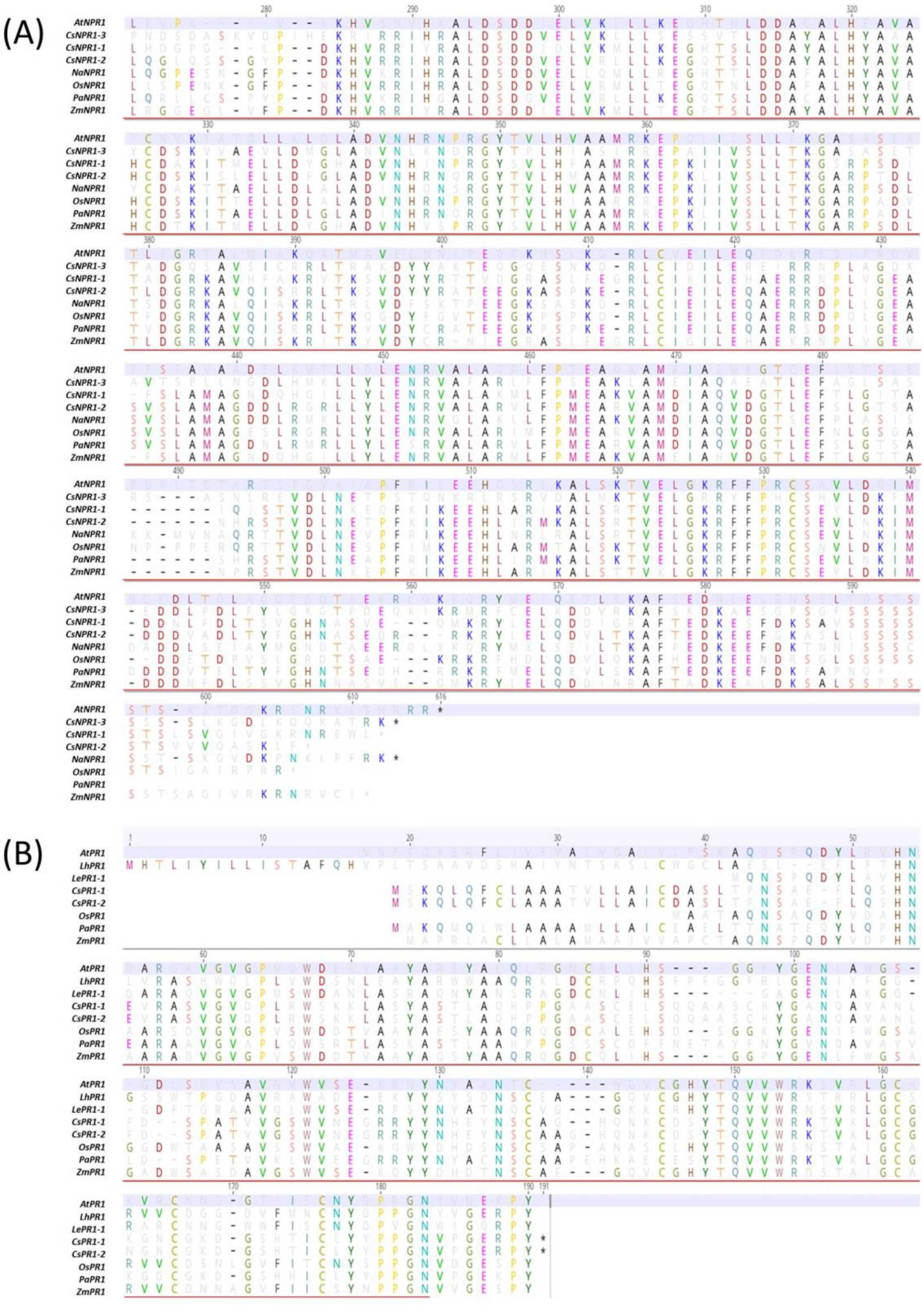

Figure S2: Alignment of deduced amino acid sequence of NPR1-like and PR1-like proteins.

Table S1 *NPR1-like* homologs used in the phylogenetic analysis and sequence alignment in this study.

Species	Gene code	Gene bank accession number	CDS length (bp)
	CsNPR1-1	-	1707
Cymbidium sinense	*CsNPR1-2*	-	1668
	CsNPR1-3	-	1446
Phalaenopsis aphrodite	*PhaNPR1*	JN630802.1	1641
	OsNPR3	HM991170.1	1770
	OsNPR1	DQ450947.1	1749
Oryza sativa	*OsNPR1-1*	HM991166.1	1908
	OsNPR1-like	AY323485.1	1749
	OsNPR2	HM991169.1	1749
Zea mays	*ZmNPR1*	NM_001367877.1	1710
	AtNPR1	NM_105102.3	1782
Arabidopsis thaliana	*AtNPR1-like 3-1*	NM_001344586.1	1551
	AtNPR1-like 3-2	NM_123879.3	1761
	AtNPR4	AY785951.1	1725
Glycine max	*GmNPR1-1*	FJ418595.1	1773
	GmNPR1-2	FJ418597.1	1773
Nicotiana tabacum	*NtNPR1*	KY402167.1	1767
	NtNPR1-like	DQ837218.1	1767
Nicotiana attenuata	*NaNPR1*	EF441289.1	1767
Nicotiana glutinosa	*NgNPR1*	EU139477.1	1767
Capsicum annuum	*CaNPR1*	DQ648785.1	1749
Capsicum chinense	*CcNPR1*	AM900559.1	1569
Populus deltoides	*PdNPR2*	JF732893.1	1764
Populus tomentosa	*PtNPR4*	MF463575.1	1437
Capsella grandiflora	*CgNPR1-1*	KT163438.1	1746
Calotropis procera	*CpNPR1*	KU950443.1	1866
Brassica juncea	*BjNPR1-1*	DQ359129.3	1782
Morus alba	*MaNPR1*	JX432965.1	1746
Triticum durum	*TdNPR1*	JX424315.1	1734

Table S2 *PR1-like homologs used in the phylogenetic analysis and sequence alignment in this study.*

Species	Gene code	Gene bank accession number	CDS length (bp)
Cymbidium sinense	*CsPR1-1*	-	501
	CsPR1-2	-	504
Phalaenopsis aphrodite	*PhaPR1*	JX137044.1	507
Oryza sativa	*OsPR1*	AF306651.1	495
	OsPR1a	AJ278436.1	504
Arabidopsis thaliana	*AtPR1*	NM_127025.3	483
Brassica juncea	*BjPR1*	DQ359128.1	483
Brassica napus	*BnPR1*	U64806.1	486
Brassica oleracea	*BoPR1*	EF423806.1	486
Brassica rapa	*BrPR1*	JX110772.1	486
Capsicum annuum	*CaPR1*	AF053343.2	537
Camellia sinensis	*CasPR1*	KF527571.1	486
Cucurbita moschata	*CmPR1*	MH105818.1	594
Helianthus annuus	*HaPR1*	KR071874.1	486
Hevea brasiliensis	*HbPR1*	KR150952.1	450
Lycopersicon esculentum	*LePR1*	DQ159948.1	408
Lilium hybrid	*LhPR1*	KY365749.1	546
Morus bombycis	*MbPR1*	GU591492.1	492
Morus alba	*MaPR1*	KC453994.1	609
Nepenthes mirabilis	*NmPR1*	GQ337079.1	498
Populus szechuanica	*PsPR1*	KP109919.1	483
Triticum aestivum	*TaPR1*	HQ848391.1	492
Vitis hybrid	*VhPR1*	AB372568.1	480
Vicia faba	*VfPR1*	JQ043349.1	471
Vitis pseudoreticulata	*VpPR1-1*	GU269633.1	528
	VpPR1-2	GU269634.1	483
Vitis vinifera	*VvPR1*	AJ536326.1	486
Zea mays	*ZmPR4*	NM_001111929.2	489

Table S3. Primer sequences used in this study

	Primer name	Primer sequence（5'→3'）*
Sequencing	S-*CsNPR1*-1-F	ATGATCTACACCGCCCAAC
	S-*CsNPR1*-1-R	GAGCCACTCTCTATTCCTT
	S-*CsNPR1*-2-F	ATGGCTCGAGTTTTTCAGCA
	S-*CsNPR1*-2-R	AAATAATTTACTTGCCTGAACAAC
	S-*CsNPR1*-3-F	ATGTATGACATGAGCGATTTGGTGCC
	S-*CsNPR1*-3-R	TTATTTCCTGGTAGCCTTCTGCTG
	S-*CsPR1*-1-F	ATGTCTAAACAGCTGCAG
	S-*CsPR1*-1-R	ATAAGGTCTCTCGCCAGG
	S-*CsPR1*-2-F	ATGTCTAAACAGTTGCAG
	S-*CsPR1*-2-R	ATAAGGTCTCTCGCCAGG
qRT-PCR	Q-*CsNPR1*-1-F	TACACTGGGCATTACTGCTATC
	Q-*CsNPR1*-1-R	TGTCCAACAGAAGTCAAGTCAAA
	Q-*CsNPR1*-2-F	ACCATAGGAACCAAAGGGGATA
	Q-*CsNPR1*-2-R	GCCATTGCCAAGGAAACAG
	Q-*CsNPR1*-3-F	AGCACGCAAAATAAAAGGC
	Q-*CsNPR1*-3-R	AGAACAGGTCAGGCAAATCA
	Q-*CsPR1*-1-F	ATTCCTTCAGTCCCACAACG
	Q-*CsPR1*-1-R	CACCCACGATCCCACCA
	Q-*CsPR1*-2-F	GCATCCTGCGAGTTGTCC
	Q-*CsPR1*-2-R	CATCCTAACTTTACCGTCTTCC
Subcellular localization	PAN-*CsNPR1*-1-F	<u>CTTAAGTCCGGAGCTAGCTCTAGAG</u>ATGATCTACACCGCCCAAC
	PAN -*CsNPR1*-1-R	<u>TCGCCCTTGCTCACCATGGATCC</u>GAGCCACTCTCTATTCCTT
	PAN -*CsNPR1*-2-F	<u>CTTAAGTCCGGAGCTAGCTCTAGAG</u>ATGGCTCGAGTTTTTCAGCA
	PAN -*CsNPR1*-2-R	<u>TCGCCCTTGCTCACCATGGATCC</u>AAATAATTTACTTGCCTGAACAAC
	PAN -*CsNPR1*-3-F	<u>CTTAAGTCCGGAGCTAGCTCTAGAG</u>ATGTATGACATGAGCGATTTGGTGCC
	PAN -*CsNPR1*-3-R	<u>TCGCCCTTGCTCACCATGGATCC</u>TTTCCTGGTAGCCTTCTGCTG
	PAN -*CsPR1*-1-F	<u>CTTAAGTCCGGAGCTAGCTCTAGAG</u>ATGTCTAAACAGCTGCAG
	PAN -*CsPR1*-1-R	<u>TCGCCCTTGCTCACCATGGATCC</u>ATAAGGTCTCTCGCCAGG
	PAN -*CsPR1*-2-F	<u>CTTAAGTCCGGAGCTAGCTCTAGAG</u>ATGTCTAAACAGTTGCAG
	PAN -*CsPR1*-2-R	<u>TCGCCCTTGCTCACCATGGATCC</u>ATAAGGTCTCTCGCCAGG
qRT-PCR + RT-PCR	CsUBQ-F	CCGGATCAGCAAAGGTTGA
	CsUBQ-R	AAGATTTGCATCCCTCCCC

* The sequences on the lines are the fusion sequence.
